# Adaptation in a keystone grazer under novel predation pressure

**DOI:** 10.1098/rspb.2024.1935

**Published:** 2025-01-22

**Authors:** Danai Kontou, Andrew M. Paterson, Elizabeth J. Favot, Christopher Grooms, John P. Smol, Andrew J. Tanentzap

**Affiliations:** ^1^ Ecosystems and Global Change Group, Department of Plant Sciences, University of Cambridge, Cambridge CB2 3EA, UK; ^2^ Dorset Environmental Science Centre, Environmental Monitoring and Reporting Branch, Ontario Ministry of the Environment and Climate Change, Dorset, Ontario P0A 1E0, Canada; ^3^ Vale Living with Lakes Centre, Cooperative Freshwater Ecology Unit, Laurentian University, Sudbury, Ontario P3E 2C6, Canada; ^4^ Paleoecological Environmental Assessment and Research Lab, Department of Biology, Queen’s University, Kingston, Ontario K7L 3N6, Canada; ^5^ Ecosystems and Global Change Group, School of Environment, Trent University, Peterborough, Ontario K9L 0G2, Canada

**Keywords:** Daphnia, predation, adaptation, invasive species, freshwater ecology

## Abstract

Understanding how species adapt to environmental change is necessary to protect biodiversity and ecosystem services. Growing evidence suggests species can adapt rapidly to novel selection pressures like predation from invasive species, but the repeatability and predictability of selection remain poorly understood in wild populations. We tested how a keystone aquatic herbivore, *Daphnia pulicaria*, evolved in response to predation pressure by the introduced zooplanktivore *Bythotrephes longimanus*. Using high-resolution ^210^Pb-dated sediment cores from 12 lakes in Ontario (Canada), which primarily differed in invasion status by *Bythotrephes*, we compared *Daphnia* population genetic structure over time using whole-genome sequencing of individual resting embryos. We found strong genetic differentiation between populations approximately 70 years before versus 30 years after reported *Bythotrephes* invasion, with no difference over this period in uninvaded lakes. Compared with uninvaded lakes, we identified, on average, 64 times more loci were putatively under selection in the invaded lakes. Differentiated loci were mainly associated with known reproductive and stress responses, and mean body size consistently increased by 14.1% over time in invaded lakes. These results suggest *Daphnia* populations were repeatedly acquiring heritable genetic adaptations to escape gape-limited predation. More generally, our results suggest some aspects of environmental change predictably shape genome evolution.

## 1. Introduction

How organisms adapt to different stressors—either natural or anthropogenic—is key for predicting future ecosystem change [1]. Growing evidence suggests that adaptation by natural selection can arise within just a few generations after drastic shifts in environmental conditions [2–5]. Genetic diversity is an important determinant of evolutionary potential to adapt to environmental change [6]. Standing genomic variation within populations serves as the substrate for selection [7]. Therefore, populations that maintain a more diverse genetic structure over time should have an increased likelihood of survival as environments gradually change [8,9] or following sudden environmental stress [10,11]. However, surviving environmental change or stressors (e.g. invasive species, heatwaves and abrupt habitat fragmentation) can lead to demographic bottlenecks where natural populations are depleted, with lower genetic diversity and drastically altered population structure that ultimately reduce fitness [12–14].

North temperate lakes are highly susceptible to multiple stressors, but relatively little is known about how they will adapt to future environmental change [15]. In the last century, many north temperate lakes have experienced biological invasions that threaten the valuable ecosystem services they provide, such as clean drinking water and food provision [16]. Although case studies explore the ecological and socio-economic impacts of invasions in lake food webs [17–19], few examine the adaptive potential of affected organisms at the genomic level. Those studies that do include genomic data about lake food webs either study a single lake or pools of individuals [20–25]. Only by sampling individuals from across a diversity of lakes will any results about the repeatability of adaptation best generalize across different environments.

Here we tested how the population genetic structure and diversity of a keystone grazer, *Daphnia pulicaria*, changed after a novel predator invaded a landscape of freshwater lakes. The water flea *D. pulicaria* (Cladocera: Anomopoda) is widely distributed across North American lakes and is part of the *Daphnia pulex* species complex, an established ‘eco-genomic’ model [26]. *Daphnia pulicaria* also appears more sensitive to anthropogenic stressors like invasive predators [27] compared with other daphniids, making it an excellent model to study how freshwater communities will eventually respond to a changing environment. Like other *Daphnia* species, *D. pulicaria* annually alternates between asexual and sexual reproductive phases (i.e. cyclical parthenogenesis [28]). The latter phase produces diploid embryos (resting stages) enclosed in protective chitinous structures called ephippia which are released with moulting and buried into sediment [29]. Up to 4000 dormant embryos per square metre of sediment surface can be produced annually [30] resulting in a rich sediment record. Alongside other exoskeletal material (e.g. carapaces, mandibles and tail spines), this record can be used to reconstruct changes in community composition and trait evolution through time [31–33].

In North America, native populations of *D. pulicaria* have been decimated by the introduction of the spiny water flea *Bythotrephes longimanus* (Cladocera: Cercopagididae) from central Eurasia, presumably through ballast waters of cargo ships in the 1980s [19,34,35]. *Bythotrephes* is highly resistant to predation [36] and can consume >30% of resident zooplankton production [37]. In lakes where *Bythotrephes* has established, total invertebrate predation can subsequently rise up to 300% [38]. Although *Bythotrephes* can coexist with planktivorous fish [39], it can displace and outcompete native predators and prey [40–43]. Thus, *Bythotrephes* not only alters species composition and key trophic interactions between them [44,45] but indirectly affects water clarity and nutrient availability by removing native grazers that normally control algal growth [19,35]. For these reasons, *Bythotrephes* should exert strong selective pressure on *D. pulicaria* populations to evade predation [46–48]. Despite observations of *Bythotrephes*-induced behavioural [49] and morphological [50] changes in populations from invaded lakes, the genetics underlying these responses have, to our knowledge, never been investigated.

In this study, we tested the hypothesis that the population genetic structure of *D. pulicaria* has changed over the last century under predation by *Bythotrephes*, and that these changes corresponded with phenotypic change. Our study design was like a ‘natural evolution experiment’, allowing us to identify the repeatability of adaptive responses. Specifically, we compared multiple environmentally similar yet spatially disconnected lakes (i.e. replicates), where some lakes were invaded and others were never invaded by *Bythotrephes*. We sampled individual *D. pulicaria* embryos from ephippia deposited in dated sediment cores. DNA can be preserved in unhatched resting embryos for centuries [31,51], and just five individuals are sufficient to reconstruct past population structure accurately [52]. Because ephippia develop under the mature female’s carapace, their length can also be used to estimate adult size [53,54], and thereby link genetic information stored in resting embryos to phenotypic traits. Although wild *Daphnia* populations can demonstrate remarkable genetic stability and low diversity [20,52], we predicted distinct changes in morphology and genetic population structure following bottlenecks coinciding with the establishment of *Bythotrephes* in invaded lakes. We expected these changes because rapid adaptive responses in *Daphnia*, such as changes in body size and fecundity, can be common under intense predation [27,47,55,56]. Together, our results provide evidence that individuals undergo similar responses to predation at a genomic level across distinct wild populations, suggesting that the spread of invasive species may leave predictable imprints on the genomes of the communities that they invade.

## 2. Methods

### (a) Lake sampling

We sampled 12 lakes with similar limnological characteristics between October and November 2021 within the District of Muskoka and Haliburton County, Ontario, Canada. The lakes were selected from a wider group of 38 lakes based on records of *B. longimanus* presence from government monitoring data and five key limnological variables known to affect the composition of plankton communities [57–59]: maximum depth, pH, and concentrations of dissolved organic carbon, total phosphorus and calcium (electronic supplementary material, figure S1). Seven lakes were first reported to have been invaded by *Bythotrephes* in 2002−2005 and five have never been invaded and were chosen as controls (electronic supplementary material, table S1). We further confirmed that chosen invaded and control lakes had similar depths and surface water chemistry at the time of sampling (electronic supplementary material, figure S2). All lakes also had similar zooplanktivore communities, comprised primarily of smallmouth bass (*Micropterus dolomieu*), yellow perch (*Perca flavescens*), trout (*Salvelinus* spp.), *Leptodora kindtii* and *Chaoborus* spp. (electronic supplementary material, table S2).

We retrieved four sediment cores (approx. 30 cm length; 7.6 cm diameter) from the deepest point in each lake basin following bathymetric maps and using a gravity corer [60] from anchored canoes. Three cores were transported to Dorset Environmental Science Centre (Ontario, Canada) and sectioned using an extruder [61] at a resolution of 2 cm for isolating zooplankton remains. Sediment slices were immediately placed into sterile Whirl-Pak bags, and tools were cleaned between increments with deionized water. One core per lake was sectioned at a finer resolution of 0.5 cm for paleo-reconstructions and ^210^Pb gamma dating. Control and invaded lakes were sampled and sectioned on different days to avoid cross-contamination. All sediment samples were stored in the dark at 5°C until further processing.

Sediment underwent ^210^Pb gamma dating following Schelske *et al.* [62]. Midpoint intervals (0.5 cm) from core sections were weighed, freeze-dried, ground to a fine powder, transferred into vials sealed with epoxy resin and then allowed to rest in the dark for a minimum of 14 days to achieve ^226^Ra and ^214^Bi secular equilibrium. The activities of radioisotopes ^210^Pb, ^137^Cs and ^214^Bi were measured using an Ortec high-purity Germanium gamma spectrometer (electronic supplementary material, figure S3). Core chronologies were established from isotope activities and sedimentation rate profiles in each lake according to the ‘Constant Rate of Supply’ model [63], and results were analysed using ScienTissiME (ScienTissiME, Barry’s Bay, Canada; (electronic supplementary material, figure S4).

### (b) Isolation of zooplankton remains from sediment

We isolated *Daphnia* ephippia and *Bythotrephes* caudal spines from sediment based on size and described morphological characteristics [64–66] (electronic supplementary material, figure S5). Sediment slices from two cores per lake were individually filtered through separate 150 μm metal sieves for control and invaded lakes using dechlorinated water to reduce osmotic stress on resting embryos [67]. Filtrates were stored at 5°C until transferred to clean Petri dishes with dechlorinated water and examined under a stereo microscope. We counted the number of *D. pulicaria* ephippia and *Bythotrephes* spines recovered from each filtrate to estimate abundance at various time-points [68,69].

To test the effect of *Bythotrephes* invasion on *Daphnia* population genetic structure, we extracted ephippia from the filtrates of six invaded and three control lakes. Cores from these lakes contained well-preserved material from which DNA could be extracted, that is, they contained completely closed ephippia with unhatched embryos. Guided by the ^210^Pb chronology for each lake (electronic supplementary material, figure S4), we selected two populations of at least six embryos [52] from the top 2 cm and a deeper 2 cm interval of each core (between 10 cm and 20 cm depth depending on the lake). The deeper (‘bottom’) sections were chosen to correspond with the onset of *Bythotrephes* appearance in the sediment record (electronic supplementary material, figure S6). Previous studies have suggested *Bythotrephes* may establish decades [69] or even centuries [70] before first detection in the water column, which only corresponds with high densities of several million individuals [71]. Consequently, the top and bottom sections, respectively, corresponded to ‘modern’ (*ca* 2010−2020) and ‘historic’ (*ca* 1900−1940) *Daphnia* populations (electronic supplementary material, figure S4).

### (c) DNA extraction and sequencing

Sample preparation for resting egg whole-genome sequencing followed a combination of established protocols [67,72]. Ephippia were dissected with sterile needles and forceps under a stereo microscope. Extrinsic membranes were removed and only one embryo per ephippium was extracted (electronic supplementary material, figure S5). Visibly degraded and misshapen embryos were discarded. Each embryo was immediately transferred in a drop of 0.5% bleach solution onto Petri dishes using a sterile pipette tip and incubated for 1 min at room temperature for decontamination. Individual embryos were rinsed in pure nuclease-free water by three consecutive tube transfers and ultimately crushed against the walls of a sterile Eppendorf DNA LoBind tube using a pipette tip. Genomic DNA was extracted from each embryo in separate tubes using a MasterPure Complete DNA and RNA Purification Kit (LGC Biosearch Technologies, Middlesex, UK), following the manufacturer’s protocol for animal tissue DNA isolation. Purified genomic DNA was resuspended in 30 μl of TrisHCl (10 mM) solution and stored at −20°C. Overall, we extracted DNA from a total of 108 individual embryos, of which 52 and 45 were from the tops and bottoms of cores, respectively, and were of sufficient DNA quantity and quality for sequencing.

DNA libraries were prepared and purified for each individual resting embryo using an Illumina DNA Prep Kit (Illumina, San Diego, USA) and following the manufacturer’s instructions for low DNA input. Libraries were tagged using unique indexes and quantified with a Qubit 3.0 high-sensitivity assay for dsDNA (ThermoFisher, Waltham, USA). Library quality was assessed with a 2100 Bioanalyzer (Agilent, Santa Clara, USA) prior to normalization, and libraries were pooled at equimolar concentrations. Libraries for each resting embryo were separately sequenced at 2 × 150 bp on an Illumina NovaSeq 6000 platform by Novogene UK.

### (d) Bioinformatics

Demultiplexed sequences were checked with FastQC v.0.11.8 (https://www.bioinformatics.babraham.ac.uk/projects/fastqc/) for GC content, sequence duplication, adapter contamination and base call quality distribution. We used fastp v.0.23.4 [73] to remove polymerase chain reaction (PCR) duplicates, trim adapter sequences, poly-G and poly-A tails, and filter out reads with a quality score <30 and shorter than 30 bp post-trimming. Filtered sequences were aligned against the latest (Feburary 2022) *D. pulicaria* reference genome assembly SC_F0−13Bv2 (GenBank accession no. GCA_021234035.2) using BBMap v.38.90 [74]. Mapped sequences for each lake with a mapping quality >20 (mean ± s.e.: 386 413 ± 190 692 and 359 855 ± 108 031 paired reads per sample from top and bottom sections, respectively) were sorted into bam files and indexed with Samtools v.1.19 [75]. We called variants from sorted bam files at a maximum depth of 10 000 sequences per position using mpileup from BCFtools v.1.18 [76]. Variants were filtered further to remove indels and single-nucleotide polymorphisms (SNPs) with low-quality scores (<20) and those with minor allele frequencies <0.05. Mean genome coverage post-filtering ± s.e. was 0.7 times ± 1.3, which was expected given the age and origin of our samples [77]. To test for bias in genome coverage, we fitted a linear mixed effects model with lme4 v.1.1−35.1 [78] in R v.4.2.1 (84). The model compared coverage among chromosomes and between both top versus bottom depths and invaded versus control lakes, accounting for random variation due to repeated measurement of the same individual.

### (e) Population genetic analyses

To explore patterns of temporal and spatial population genetic structure of *D. pulicaria* following the introduction of *Bythotrephes*, we fitted a Principal Coordinate Analysis (PCoA) with the cmdscale R function [79]. A matrix of identity-by-state genetic distances between individuals from core tops and bottoms was generated with PLINK v.1.90b5.3 [80] from a normalized vcf file containing bi-allelic SNPs for all nine lakes from which we sequenced resting embryos. Individuals with >50% missing data were removed prior to the PCoA. We further tested if contemporary *D. pulicaria* populations were increasingly differentiated with the geographic distance between lakes using a Mantel test of both genetic and spatial distance matrices with R package ade4 v.1.7−22 [81].

To test how *D. pulicaria* population structure changed after the establishment of *Bythotrephes*, we first generated genotype likelihood files for each individual from both invaded and control lakes using ANGSD v.0921 [82]. Genetic structure was inferred with an iterative algorithm in NGSadmix v.32 [83], where individuals were grouped into distinct clusters based on shared variation. The method is similar to that implemented in STRUCTURE [84] but better suited to low-coverage, next-generation sequencing data like ours [83]. To identify the probable number of distinct genetic clusters (*K*) in each lake at each time point, we ran NGSadmix 10 times for every *K* from 1 to 8 [84,85]. The best supported *K* for each lake was that which resulted in the greatest increase in model likelihood between successive *K*-values [85]. Replicate runs from the best-supported *K* were then concatenated into a probability matrix of cluster membership for each sample using CLUMPP v.1.1.2 [86].

For each lake, we also compared genome-wide nucleotide diversity (*π*) and genetic differentiation (*F*_ST_) between individuals and populations. Estimates for both metrics were generated over 5 kb sliding windows at 1 kb steps using VCFtools v.0.1.16 [87]. We fitted a linear mixed effects model with lme4 v.1.1−35.1 in R [78] to compare nucleotide diversity between top and bottom populations within each lake and between invaded and uninvaded lakes at each time-point. For *F*_ST_ calculations, VCFtools implements the method of Weir & Cockerham [88], which corrects for differences in allele frequency distributions between populations while accounting for sample size. This approach is considered more robust for small or varied sample sizes like ours [89]. Pairwise *F*_ST_ values between top (‘modern’) and bottom (‘historic’) populations were then calculated with the R package StAMPP v.1.6.3 [90]. This approach uses bootstrapping across loci during calculations to test if populations were differentiated more than expected by chance, i.e. are statistically different, and *F*_ST_ differences were considered significant after a Bonferroni correction accounting for multiple comparisons (*α* < 0.001).

Finally, to identify potential signatures of selection by *Bythotrephes* predation, we examined regions of elevated genomic differentiation before (bottom) and after (top) the invasion. Loci with a mean pairwise *F*_ST_ value falling within the top 1% of the genome wide *F*_ST_ estimates across all invaded and uninvaded lakes were marked as outliers. We then extracted all 5 kb windows centred on outlier loci. For invaded lakes Grandview and Fletcher, where mean outlier numbers per chromosome were large (60 and 513, respectively), only windows with the highest density of outliers (>10) were considered. More conservative per-locus *F*_ST_ estimates and their respective probabilities of being under selection were calculated with a Bayesian likelihood approach as implemented in BayeScan v.2.01 [91]. Using the generated sets of quality-filtered SNPs per lake, we first applied linkage disequilibrium (LD) pruning with a threshold of 0.2 and excluded all monomorphic loci to reduce redundancy using BCFtools v.1.18 [76] (electronic supplementary material, table S4). We then ran BayeScan with default model parameters for 20 pilot runs with 2000 iterations, 50 000 burn-ins followed by 50 000 iterations and prior odds of the neutral model set to 10.

Positions within the selected 5 kb windows and any loci with elevated *F*_ST_ identified by BayeScan were cross-referenced against a curated list of protein coding genes associated with *Daphnia* embryo development, body size and carapace strength that are known to be expressed differentially after exposure to predator cues (electronic supplementary material, table S5 and references therein). The same outlier positions were also cross-referenced against the Ensembl Genome Browser to extract information about the molecular and biological functions of any previously annotated loci [92] (electronic supplementary material, table S6). We also inspected positions up to 10 kb upstream and downstream of the identified 5 kb windows, as these often include regulatory regions [21,93] but never detected any outliers.

### (f) Ephippial size analysis

To identify phenotypic changes in *Daphnia* that accompanied genetic differentiation in lakes invaded by *Bythotrephes*, we measured 2288 ephippia isolated from sediment. All complete *D. pulicaria* ephippia from each sediment interval were isolated and photographed with a GXCAM-U3−5 microscope camera (GT Vision, Suffolk, UK). Length along the dorsal ridge was manually measured for each ephippium using ImageJ [94] (electronic supplementary material, figure S5b). We tested if mean ephippial length changed over time in lakes invaded by *Bythotrephes* with a linear mixed effects model fitted using lme4v.1.1−35.1 in R [78]. Ephippial lengths were log-transformed, and both *Bythotrephes* presence and core section were included as fixed factors in our model. We accounted for repeated measurements in the same lake by including lake identity as a random effect.

## 3. Results

### (a) Population genetic structure across time and space

**Figure 1.**
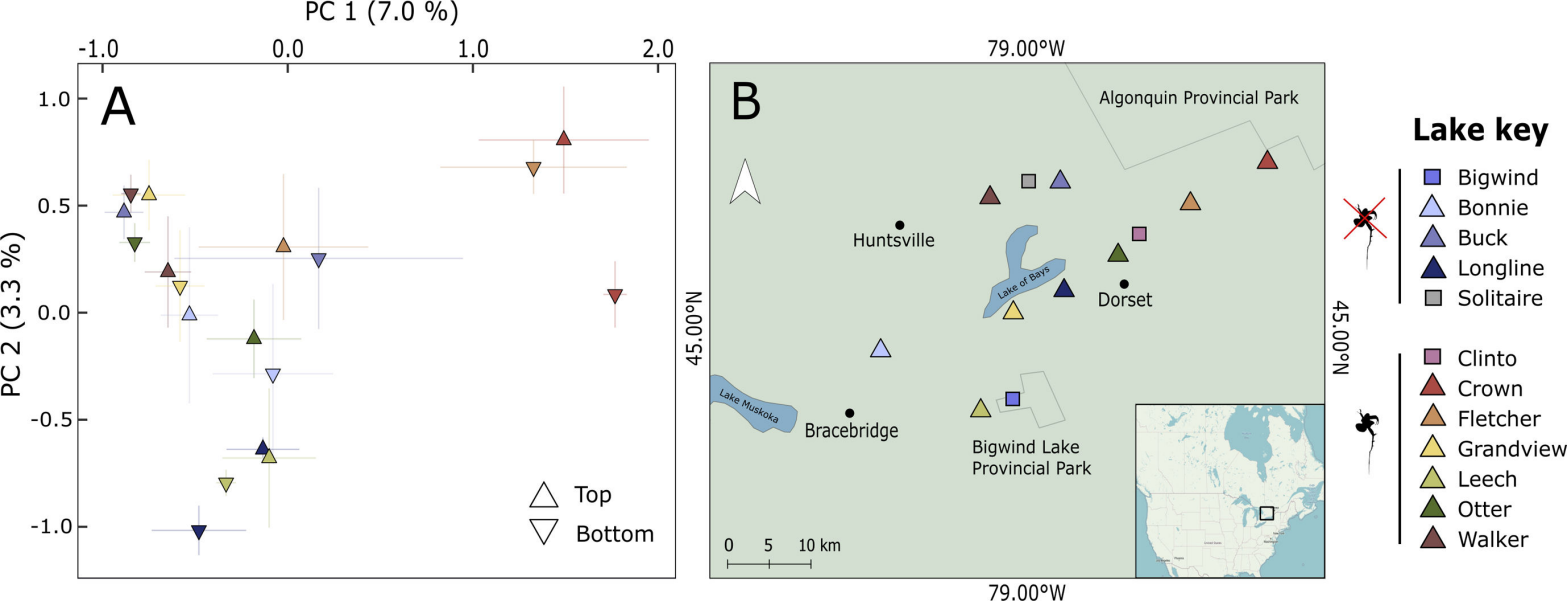
Evidence of spatial and temporal genetic structure in *Daphnia pulicaria* across central Ontario, Canada. (A) PCoA of genetic distance between individual embryos across time and space. Points are centroids ± standard error for individual resting embryos (*n* = 9–13 per lake) within ‘modern’ (top) and ‘historic’ (bottom) populations in each lake separated by approximately 100 years. (B) Regional map of lakes. Triangles mark lakes where both phenotypic and genomic data were collected (*n* = 9) and squares mark lakes with only phenotypic data (*n* = 3) due to a lack of preserved embryos suitable for DNA extraction.

We found evidence of temporal and spatial population genetic structure between *D. pulicaria* populations, consistent with our expectation that predation by the introduced *B. longimanus* exerts a strong selective pressure on its prey. ‘Modern’ (top) and ‘historic’ (bottom) subpopulations were separated in the PCoA with the largest differences often observed within invaded lakes (figure 1A). However, ‘modern’ subpopulations were visually separated in the PCoA along a west-to-east gradient from the townships of Bracebridge (Leech and Bonnie lakes) and Lake of Bays (Grandview, Longline, Otter and Walker lakes) to Huntsville and the Algonquin Highlands in Haliburton County (Buck, Crown and Fletcher lakes; figure 1B). In support of this interpretation, *Daphnia* populations from lakes that were more geographically distant from each other were more genetically differentiated (Mantel test, *r* = 0.40, *p* < 0.001).

**Figure 2.**
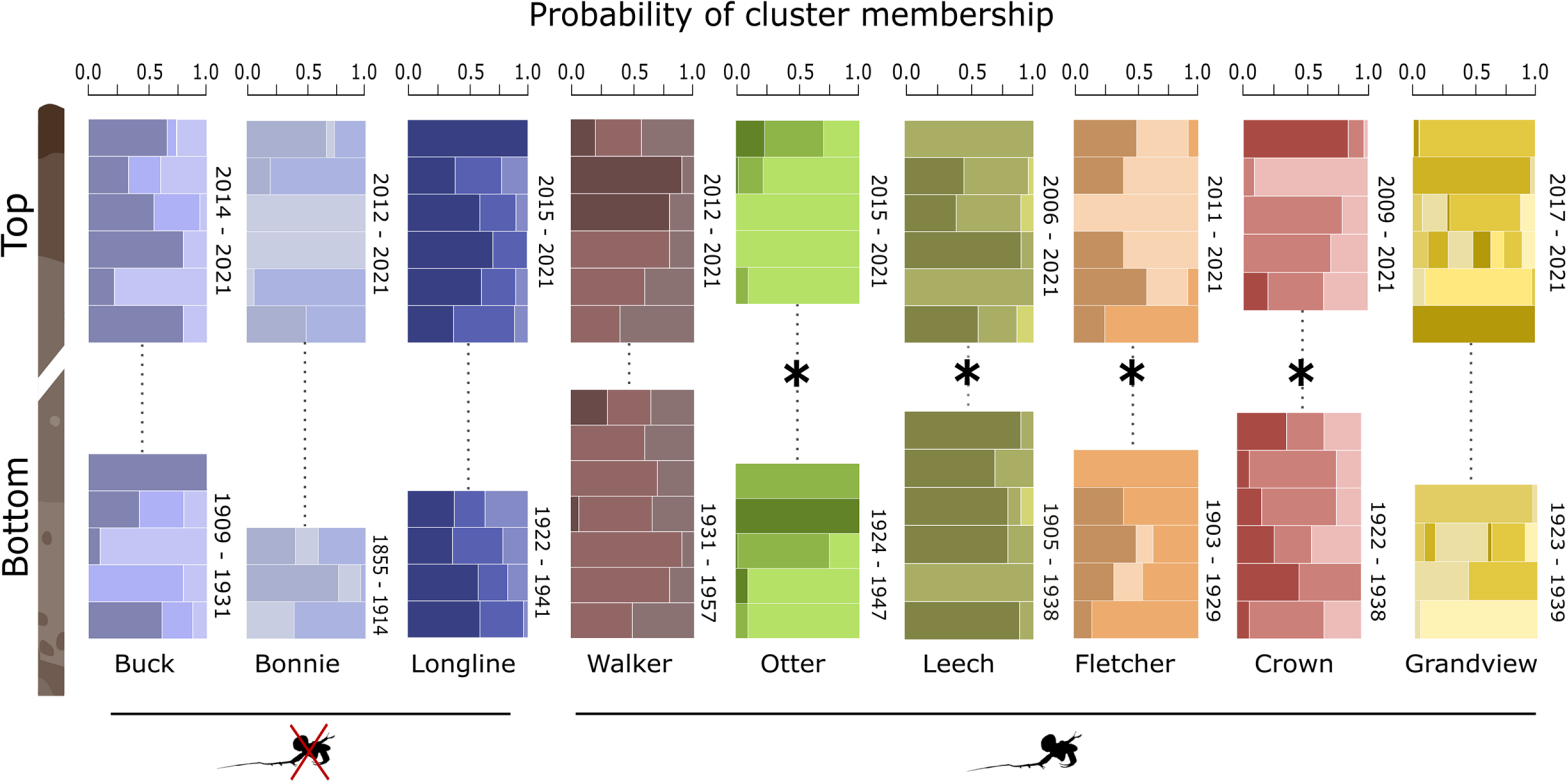
Population genetic structure changed between modern (core top) and historic (core bottom) resting embryos in four lakes invaded by *Bythotrephes*. Individual embryos from each lake core top and bottom were classified into three (*K* = 3) or seven (*K* = 7) potential genetic clusters based on maximum genotype likelihoods estimated by NGSadmix [83]. Each horizontal bar represents an individual embryo with a sequenced genome, and each unique colour shade marks a distinct genetic cluster. Colour proportions reflect the probability of an individual belonging to a respective cluster. Asterisks mark lakes where we observed statistically significant genomic differentiation (*F*_ST_ ≥ 0.08, *p* < 0.001) after approximately 100 years, and these changes only occurred in lakes where *Bythotrephes* was introduced.

Genetic structure within populations changed after approximately 100 years in four of the six invaded lakes but in none of the uninvaded lakes, according to our admixture analysis. Although we recovered few unique genetic clusters (*K* = 3) in all but one lake (Grandview, *K* = 7), the proportion of these clusters changed over time in invaded Crown, Fletcher, Leech and Otter lakes, as estimated by mean pairwise genetic differentiation (figure 2). In these four invaded lakes, we observed both low (Otter *F*_STp_ = 0.08 and Crown *F*_STp_ = 0.10) and very high (Leech *F*_STp_ = 0.58 and Fletcher *F*_STp_ = 0.91) genetic differentiation following the introduction of *Bythotrephes,* whereas all ‘modern’ and ‘historic’ subpopulations from all three controls and two invaded lakes (Grandview and Walker) could be considered fully admixed (*F*_STp_ = 0.0; table 1). In the two highly differentiated, invaded lakes (Fletcher and Leech), *Bythotrephes* caudal spines appeared deeper in the sediment (year ± standard error: 1923 ± 10 in Leech, 1930 ± 4 in Fletcher) compared with the other invaded lakes (1958 ± 2 in Otter, 1966 ± 2 in Crown, 1941 ± 2 in Walker and 1976 ± 2 in Grandview). These results suggest the extent of genetic differentiation may increase with the length of time that populations are exposed to *Bythotrephes*, though the density of spines was almost 200% lower in historic sediments than in core tops (electronic supplementary material, figures S4 and S6A).

**Table 1.** Greater genetic differentiation over time in *Daphnia pulicaria* populations from lakes invaded by *Bythotrephes*. We identified SNPs from 9 to 13 individual embryos (*n*) in each of the nine lakes in Ontario, Canada. We used the individual embryos to calculate genetic differentiation (*F*_ST_) between modern (core tops) and historic (core bottoms) populations in each lake. In Longline Lake, there was insufficient overlapping sequencing coverage to calculate pairwise *F*_ST_ between core sections. n.s. = not statistically significant. ***p < 0.001 after a Bonferroni correction for multiple comparisons.

lake	status	*n*	SNPs	*F*_ST_ (top versus bottom)	
Bonnie	control	9	21	0.00	n.s.
Buck	control	11	548 411	0.00	n.s.
Longline	control	11	269 925	—	—
Crown	invaded	11	67 613	0.10	***
Fletcher	invaded	11	257 782	0.91	***
Grandview	invaded	10	220 785	0.00	n.s.
Leech	invaded	12	11 271	0.58	***
Otter	Invaded	10	48 078	0.08	***
Walker	invaded	13	545 366	0.00	ns

### (b) Genomic differentiation in lakes invaded by *Bythotrephes*

**Figure 3.**
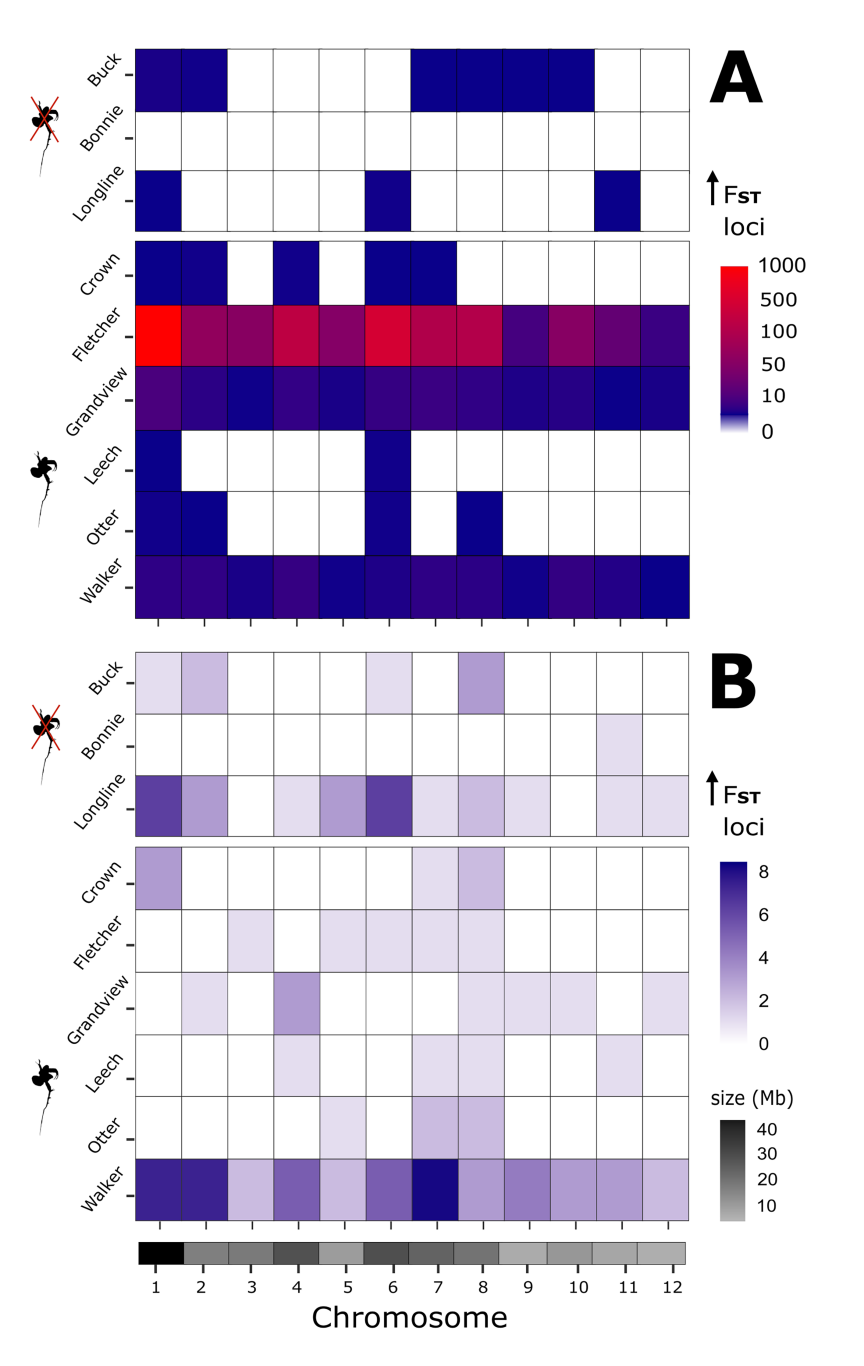
Genomic differentiation after approximately 100 years in contemporary *Daphnia pulicaria* populations from lakes invaded by *Bythotrephes*. (A) Number of loci marked as statistical outliers defined as a positive *F*_ST_ value in the top 1% of the genome-wide estimate for each lake. Weir & Cockerham *F*_ST_ was estimated for 5 kb intervals at 1 kb over the entire genome, comparing individual genotypes between core tops (‘modern’: 2010−2020; *n* = 5–6 genotypes per lake) and bottoms (‘historic’: 1920−1940; *n* = 3–7 genotypes per lake). (B) Distribution of outlier loci with elevated *F*_ST_ estimated by BayeScan v.2.1. Outliers in invaded lakes included loci within annotated RNA and protein coding regions associated with development, physiology or environmental stress responses (electronic supplementary material, tables S5 and S6). Chromosome lengths (Mb) displayed in greyscale below.

We found evidence of stronger genome-wide differentiation between core tops and bottoms in *D. pulicaria* populations from the invaded lakes but not the controls. Almost 99% of all biallelic SNPs with *F*_ST_ values in the top 1% of genome-wide estimates were observed in invaded lake genotypes (figure 3A). The proportion of windows containing *F*_ST_ outliers ranged from 0% to 10% in uninvaded controls and 2% to 16% in invaded lakes, except for Fletcher, where the percentage was higher (75%). Only 0.04% of windows contained outliers in uninvaded Buck Lake despite having almost twice as many windows covered than invaded Fletcher Lake (electronic supplementary material, table S4), suggesting there was no sequencing coverage bias. BayeScan detected 1–57 loci per lake with elevated *F*_ST_ but none were marked as statistically significant (figure 3B).

Consistent with elevated *F*_ST_, we found further evidence of greater genetic diversity where *Bythotrephes* was present, potentially due to increased investment in sexual reproduction under predation pressure (electronic supplementary material, figure S8). Genome-wide nucleotide diversity (*π*) decreased over time across all sites but was 49.4% higher within populations from invaded lake tops than within contemporary controls (*t* = 96.8, d.f. = 14 400, *p* < 0.001; electronic supplementary material, figure S8). Our observations were not simply due to differences in sequencing coverage. Although individual coverage ranged from 0.2 to 10 times depending on sample quality, mean coverage was similar across all lakes irrespective of *Bythotrephes* invasion and among all 12 chromosomes (electronic supplementary material, figure S7 and table S3).

Focusing on areas within the *D. pulicaria* genome with elevated *F*_ST_ between pre- and post-establishment of *Bythotrephes*, we found evidence of potential adaptation of *D. pulicaria* to increased predation pressure. We detected a similar pattern of genome differentiation in invaded lakes, but no statistically significant evidence for selection of individual loci. We identified 147 windows unique to the invaded lakes, where at least 1−3 outlier loci clustered within a single 5 kb region of elevated *F*_ST_ that was also associated with development, physiology and environmental stress responses (figure 3; electronic supplementary material, figure S9). Of these 147 windows spread across seven chromosomes, eight windows (in Grandview and Fletcher lakes) and one locus identified by BayeScan (in Walker Lake) fell within our curated list of protein-coding genes with known differential expression in response to predation and stress (electronic supplementary material, table S5). All these genes have been linked to embryo development, body size, ephippia production, carapace strength and inducible defences in experimental populations of congeneric species *D. pulex* and *Daphnia magna* (electronic supplementary material, table S5). The other 139 windows with outliers aligned approximately within 200 kb of each other when on the same chromosome and were distributed throughout the genome (electronic supplementary material, figure S9 and table S6).

There was no overlap of outlier windows between invaded and control lakes. Physical overlap was only observed on chromosome 1, with five windows between three invaded lakes (Fletcher, Walker and Grandview) and six windows across chromosomes 2, 5 and 8 within 20 kb from each other (Fletcher and Walker; electronic supplementary material, figure S9 and table S6). Outlier windows included at least 40 protein-coding genes associated with development and body size (e.g. nervous and muscle tissue, biosynthetic and metabolic pathways of collagen and lipids), with another 60 genes potentially involved with physiological responses to environmental stress (e.g. protein modification, ion transport and DNA repair). Finally, 27 loci corresponded to long non-coding (lnc)RNA sequences known for their role in epigenetics, a crucial mechanism for rapid responses of *Daphnia* mothers to environmental cues, as well as sex determination, which can in turn affect resting embryo production in cyclical parthenogens like *D. pulicaria* [95,96] (electronic supplementary material, table S6). We found another 28 potential outlier positions within genes of similar functions across invaded lakes using BayeScan (electronic supplementary material, table S7).

### (c) Phenotypic response accompanies genetic differentiation

**Figure 4.**
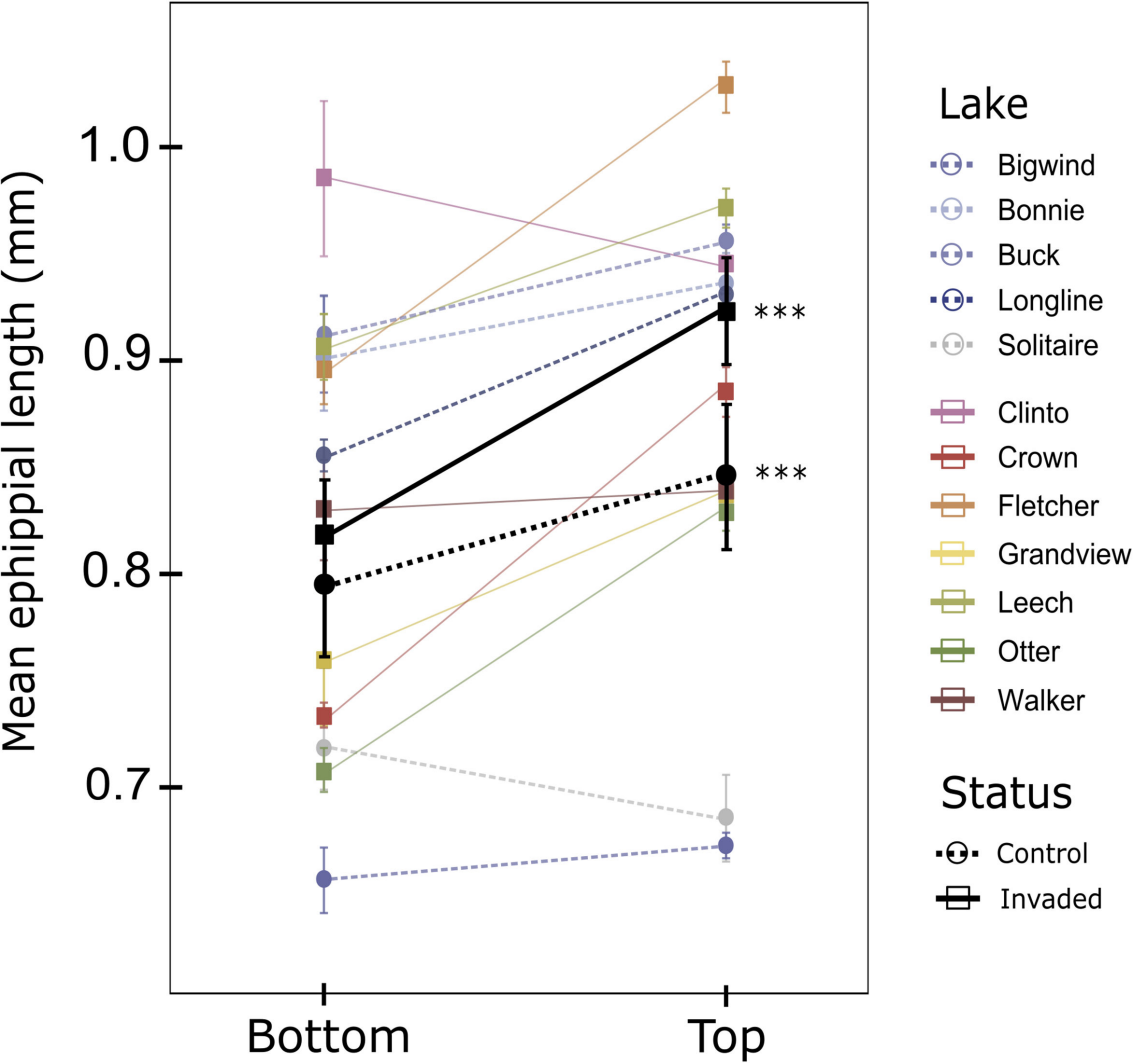
Ephippial length increased over time in lakes invaded by *Bythotrephes.* Points and whiskers show mean ephippium length ± s.e. in ‘historic’ (bottom) and ‘modern’ (top) resting embryo banks. Black lines and whiskers are mean estimated change across all seven invaded and five control lakes: mean ± s.e. of 14.1 ± 0.7% (*t* = 5.28, d.f. = 2278 and *p* < 0.001) and 6.7 ± 1.0% (*t* = 6.38, d.f. = 2278 and *p* < 0.001), respectively. Total number of ephippia (*N*) varied among sections and lakes from *N*_Bottom_ = 7–175 and *N*_Top_ = 11–506.

Consistent with the strong differentiation of genomic regions involved in development and body size, we found that the size of *D. pulicaria* ephippia predictably increased in top sediments after lakes were invaded by *Bythotrephes*. In invaded lakes, ephippial length increased from core bottoms to tops by a mean ± s.e. of 14.1 ± 0.7%, from 0.79 ± 0.04 mm to 0.91 ± 0.04 mm (core depth × invasion interaction: *t*_2278_ = 5.28, *p* < 0.001). This increase was more than twice that in the control lakes, where mean size only increased by 6.7 ± 1.0% over the same period, from 0.79 ± 0.04 mm to 0.84 ± 0.05 mm (main core depth effect: *t*_2278_ = 6.38, *p* < 0.001; figure 4). There was otherwise no difference between invaded and uninvaded lakes across both core bottoms and tops (main invasion effect: *t*_10_ = 6.38, *p* = 0.912).

## 4. Discussion

Using a natural experiment, we found that introduction of an invasive predator repeatedly changed both the genotype and phenotype of native prey populations, irrespective of their unique demographic history. Consistent with our expectations, genetic differentiation increased in multiple *D. pulicaria* populations, and genetic structure became dominated over time by genotypes with elevated differentiation in regions associated with adaptive responses to environmental stress. Previously reported *Bythotrephes* invasion dates imply these changes were relatively rapid, occurring within 30 years of intense predation pressure and accompanied by an increase in *Daphnia* body size at the time of reproduction. Although body size is also influenced by environmental conditions like temperature and nutrient availability [26,56], we controlled for these variables by sampling lakes that were physically and chemically similar within the same climate zone.

Predation by *Bythotrephes* likely caused a severe bottleneck in invaded lakes, as documented by a 20%–113% decrease in the number of *Daphnia* ephippia shortly after the appearance of *Bythotrephes* spines, despite these remains being even better preserved as they were in more recently deposited sediments (electronic supplementary material, figure S6B). Past studies have recorded similar declines (18–30%) once *Bythotrephes* invades [44,50]. Such a drastic reduction in population size would explain the different population genetic structure between time periods, as well as temporal trends of nucleotide diversity that can vary in natural populations of cyclical parthenogens such as *D. pulicaria* [97]. Although it is possible to infer bottleneck strength from sequence data, this process relies on metrics that are highly sensitive to sample size (e.g. Tajima’s D) and can be less informative for small samples like ours [98]. Nevertheless, the consistent responses to predation among lakes with different population genetic structure further suggests that these potential bottlenecks were driven by the same strong selective pressure.

Our results now offer insight into the genetic mechanisms that may accompany similar changes in population-level behaviour and morphology elsewhere novel predators have been introduced. For example, Caribbean *Anolis* lizards alter their habitat use in the presence of introduced predators by hiding in vegetation and evolving larger sizes [99]. Similarly, *Littorina* gastropods adapt to predation by changing their shell morphology to avoid gape-limited crabs [100]. In both cases, changes are attributed to a positive selection gradient, where a novel predator selects for prey that is larger or difficult to capture. This mechanism is consistent with that observed in our system, where gape-limited *Bythotrephes* selects for larger *Daphnia* body and thus embryo size by preferentially consuming smaller individuals. However, a small increase in size was also observed without *Bythotrephes* over the same period. One explanation is that increased nutrient availability, such as associated with recovery from historical acidification [101], might have contributed to consistent body size increases.

In addition to detecting phenotypic changes coinciding with genome differentiation, we identified new areas of the *D. pulicaria* genome that are potentially under selection by predation. Of 147 outlier rich loci with a known molecular function, only eight were previously associated with predation stress in *Daphnia* from studies involving experimental populations in controlled settings [102–104]. Although we observed little physical overlap among loci from invaded lakes, we detected clear functional overlap. This observation agrees with recent studies demonstrating a variety of responses to predation and other stressors, even within the same *Daphnia* populations, involving several gene families and loci with high functional redundancy [22,105]. In our study, many loci were associated with physiology, embryo development, body plasticity and maternal effects, such as muscle and neural tissue development, lipid metabolism and lncRNAs [105–107]. These functions are typically modified by complex sensory and behavioural responses to predation [108]. For example, *Daphnia* commonly migrate to deeper waters to avoid *Bythotrephes* [49]. Both membrane proteins and ion channels are crucial for daphniids to cope with different temperatures and water chemistry found at deeper depths such as by altering nutrient uptake [109,110], while certain proteinogenic amino acids like prolines are directly involved in thermoregulation and swimming behaviour [111]. It is also likely that *Daphnia* are responding to changes in their environment indirectly associated with *Bythotrephes*, such as reductions in water clarity [19,35] or taxonomic shifts in phytoplankton composition arising from predation-mediated changes in grazer communities [44].

By comparing whole genomes extracted from wild embryos instead of cultured clonal lineages, and focusing on multiple areas with elevated *F*_ST_ and outlier density, these analyses avoided biases due to *a priori* hypotheses regarding the role of candidate genes [112]. Directly sequencing resting embryos as opposed to resurrected individuals also reduces non-random selection of ancestral genotypes that often affects similar eco-evolutionary studies [113] and offers an unbiased representation of ancestral populations [52]. Although we might have overestimated the number of *F*_ST_ outliers by not controlling for physical linkages between loci and because of the high gene duplication rates in the *D. pulex* complex [26,114], the large section of the *D. pulicaria* genome that we identified as putatively under selection was consistent with the strong genome-wide differentiation that we observed post-invasion. Our outlier analysis was even possibly conservative by underestimating the actual number of overlapping windows and outliers due to relatively low genome coverage and sample size. Specifically, weak selection spread across multiple loci can be difficult to detect [115], and outlier detection with BayeScan becomes increasingly difficult as sample sizes decline [91,116]. Experimental validation and transcriptomic analyses can now help verify the exact role of identified loci in environmental stress responses and any adaptive benefits to *Daphnia*.

A major advance of our study was to interpret widespread reports of predator-induced phenotypic change alongside the underlying genetic mechanisms. For example, a study of nearby Harp Lake, Muskoka, observed an 18% reduction in Cladocera abundance and a 200% increase in mean body size within 6 years of *Bythotrephes* invasion [50]. Wathne *et al.* [117] also demonstrated that *D. pulex* clones rapidly adapted to introduced perch predation by producing 5% larger offspring that matured earlier [117]. Although we measured ephippial rather than body size, previous studies have shown that the 11% mean increase in ephippial size observed here can correspond to an almost equal increase in adult size [54,117,118]. Larger individuals can ultimately increase predator handling time [119] and escape gape-limited predators sooner by reaching a size refuge [120]. Thus, investment in larger offspring can be an advantageous evolutionary strategy for *Daphnia* and other prey species [121].

Our study provides further evidence of the complexity underlying plasticity and adaptive responses to predation in *Daphnia*. Our findings of a shared phenotypic response and increased genetic differentiation after a predator invasion across multiple lakes also expand on previous ecological and phenotypic observations [27,49,50,117,121] to improve our understanding of how keystone populations will respond to environmental change. Many daphniid populations have already been greatly reduced by *Bythotrephes* and lake water calcium declines across North America [17] and are further threatened by salinization [122], surrounding land-use changes [123] and climate warming [18]. Knowledge of the adaptive potential of populations to environmental change, like that generated here, can ultimately help prioritize efforts to conserve freshwater food webs and the vital ecosystem services that they provide.

## Data Availability

Data and code used in this study are accessible in Dryad Digital Repository [124]. Supplementary material is available online [125].
